# Changes in prenatal care and vaccine willingness among pregnant women during the COVID-19 pandemic

**DOI:** 10.1186/s12884-022-04882-x

**Published:** 2022-07-13

**Authors:** Daniel J. Erchick, Smisha Agarwal, Alexander Kaysin, Dustin G. Gibson, Alain B. Labrique

**Affiliations:** 1 Department of International Health, Johns Hopkins Bloomberg School of Public Health, 615 N. Wolfe St, Baltimore, MD 21205 USA; 2 Johns Hopkins University Global mHealth Initiative, Johns Hopkins Bloomberg School of Public Health, Baltimore, MD USA; 3 Department of Family Medicine, University of Maryland Capital Region Health, Largo, MD USA; 4 Department of Epidemiology, Johns Hopkins Bloomberg School of Public Health, Baltimore, MD USA

**Keywords:** COVID-19, Pregnancy, Inequity, Vaccine, Vaccination

## Abstract

**Introduction:**

Concerns about SARS-CoV-2 infection risk in health care settings have resulted in changes in prenatal care and birth plans, such as shifts to in-person visits and increased Cesarean delivery. These changes may affect quality of care and limit opportunities for clinicians to counsel pregnant individuals, who are at higher risk of severe COVID-19 disease and adverse pregnancy outcomes, about prevention and vaccination.

**Methods:**

We conducted a cross-sectional online survey of United States adults on changes in prenatal care, COVID-19 vaccine willingness, and reasons for unwillingness to receive a vaccine. We summarized changes in access to care and examined differences in vaccine willingness between pregnant and propensity-score matched non-pregnant controls using chi-squared tests and multivariable conditional logistic regression.

**Results:**

Between December 15–23, 2020, 8481 participants completed the survey, of which 233 were pregnant. Three-quarters of pregnant women (*n* = 186) experienced a change in prenatal care, including format of care (*n* = 84, 35%) and reduced visits (*n* = 69, 24%). Two-thirds experienced a change in birth plans, from a hospital birth to home birth (*n* = 45, 18%) or vaginal birth to a Cesarean delivery (*n* = 42, 17%). Although 40% of pregnant women (*n* = 78) were unwilling to receive COVID-19 vaccination, they had higher, though non-significant, odds of reporting willingness to receive vaccination compared to similar non-pregnant women (aOR 1.38, 95% CI: 0.95, 2.00).

**Conclusion:**

To support pregnant women through the perinatal care continuum, maternity care teams should develop protocols to foster social support, patient-centered education around infection prevention that focuses on improved risk perception, expected changes in care due to COVID-19, and vaccine effectiveness and safety.

**Supplementary Information:**

The online version contains supplementary material available at 10.1186/s12884-022-04882-x.

## Significance statement

The COVID-19 pandemic changed prenatal care and birth plans for a majority of pregnant women in the United States in 2020. Our study results contribute to limited evidence on COVID-19 related disruptions, especially reduced visits and increases in home delivery and Cesarean delivery despite no known indications for change in mode of delivery. Many pregnant women were unwilling to receive COVID-19 vaccination, primarily due to concerns about safety, effectiveness, and conspiracy theories. Pregnant women most trusted information on vaccination from the Centers for Disease Control and Prevention, their primary care doctor, or family, suggesting the importance of these stakeholders for counseling and communications interventions.

## Introduction

During the first year of the coronavirus disease 2019 (COVID-19) pandemic, concerns around risk of SARS-CoV-2 infection in health care settings resulted in obstetric care providers partly shifting routine prenatal and postnatal visits from in-person to remote [1]. To mitigate transmission risk, hospitals limited the number of in-person prenatal visits as well as the number of companions present during childbirth and, in some cases, even barred their presence entirely [2, 3]. Changes in prenatal care and uncertainty about plans for labor and delivery have implications for potential downstream adverse consequences for mothers and infants and may limit opportunities for clinicians to appropriately counsel and guide pregnant individuals about COVID-19 prevention and vaccination.

Pregnant individuals infected with SARS-CoV-2 are at an increased risk of severe illness leading to hospitalization, intensive care, mechanical ventilation, and death [4]. They may face increased risk of adverse maternal and neonatal outcomes, including stillbirth, maternal death, and maternal depression [5, 6]. This elevated risk for pregnant individuals and their infants emphasizes the need for individuals to closely follow preventive measures to protect against SARS-CoV-2 infection and consider seeking COVID-19 vaccination. Many clinical practice guidelines for management of SARS-CoV-2 infection in pregnancy have been developed, but there is need for greater consensus and promotion of evidence-based guidelines for this purpose by national and international professional societies [7].

Evidence thus far suggests that messenger RNA (mRNA) COVID-19 vaccines are efficacious in pregnant individuals and confer some protection to the fetus and infant, as vaccine-generated antibodies are detectable in umbilical cord blood and breastmilk samples [8–12]. Recently published data from V-safe Surveillance System and Pregnancy Registry suggest that the vaccine is safe during pregnancy; the proportion of adverse pregnancy and neonatal outcomes, including fetal loss, preterm birth, congenital anomalies, small-for-gestational age, and neonatal death based on the V-safe data are similar to incidences in pregnant populations before the COVID-19 pandemic [13, 14]. Given the potential benefits of vaccination and the encouraging results about vaccine efficacy and safety for pregnant individuals, several leading organizations, including the Centers for Disease Control and Prevention (CDC) and American College of Obstetricians and Gynecologists (ACOG) and the Society for Maternal-Fetal Medicine (SMFM) released early recommendations that pregnant or lactating individuals may choose to receive the COVID-19 vaccine [15–18]. On August 11, 2021, the CDC issued a recommendation that pregnant and lactating individuals should receive the vaccine [19].

Despite availability of safe and effective COVID-19 vaccines, vaccine hesitancy continues to undermine efforts to control the COVID-19 pandemic. In a global survey of pregnant individuals conducted between October and November 2020, only 52% pregnant women globally, and 45% in the United States, said they would receive a hypothetical safe and free vaccine that is 90% efficacious [20]. In a cross-sectional study of three centers in the United States between August and December 2020, only 41% of pregnant women surveyed said they would receive the vaccine [21]. Reasons for reluctance in the 2020 global survey included concerns about side effects of the vaccine, as well as safety and effectiveness data among pregnant women. Additionally, anti-vaccination campaigns have raised erroneous claims linking COVID-19 vaccines with infertility, which has exacerbated reluctance in some communities [22].

The Johns Hopkins University National Pandemic Pulse is a US population-representative, internet phone/computer survey designed to obtain data on preventive behaviors, risk perceptions, agency and stigma, and misinformation related to the ongoing COVID-19 pandemic across census regions. The aim of this study is to explore changes in prenatal care and birth planning and willingness of pregnant individuals to receive a COVID-19 vaccine using data from the second National Pandemic Pulse survey conducted in December 2020. Our goal is to generate initial insights for clinical providers and policymakers to support development of strategies to communicate with pregnant individuals about the importance of receiving COVID-19 vaccination. Given the high-risk nature of this population, slowing vaccination rates in the United States, and concerns about vaccine safety and emerging misinformation, this research is critical to efforts to increase vaccine uptake and control the COVID-19 pandemic.

## Methods

### Context and study population

We fielded a nationally representative survey of adults ages 18 and older residing in the United States from December 15–23, 2020, approximately 2 weeks after the U.S. Food and Drug Administration (FDA) issued the first emergency use authorization for a COVID-19 mRNA vaccine developed by Pfizer/BioNTech. The survey was administered by Dynata, a market research firm (https://www.dynata.com) that maintains a large first-party global data platform, including 62 million panelists with accompanying demographic information. Dynata randomly selected a sample of survey participants to match the U.S. Census estimates using demographic quotas for age, gender, race, income, and census region. Our study included all participants who reported in the survey that they were currently pregnant as well as a matched sub-sample of non-pregnant individuals identifying as women. Security and data quality checks utilized included digital fingerprinting and spot-checking via third-party verification to confirm the identity of the respondents and prevent duplication. Participants received a small compensation for survey completion.

### Questionnaire

The National Pandemic Pulse survey questionnaire included questions on participant demographic characteristics, socioeconomic status, political affiliation, and basic health information, such as type of insurance and chronic conditions (Appendix 2). Participants identifying as pregnant responded to a module of questions on COVID-19 related disruptions in prenatal care, perceived quality of care, and pregnancy-related resources adapted from the COVID-19 questionnaire by the Environmental Influences on Health Outcomes (ECHO) program supported by the National Institutes of Health [23]. We also included questions developed by our research team on COVID-19 vaccine willingness and reasons for being unwilling to receive a vaccine. Specifically, we asked participants how willing they would be to receive a COVID-19 vaccine. Participants could respond as extremely willing, willing, unwilling, or extremely unwilling. For those who reported being unwilling to receive a COVID-19 vaccine, reasons for this hesitancy were captured. Participants were also asked about which sources they trust most for vaccine-related information.

### Statistical analysis

Our analysis focused on changes in prenatal care and vaccine willingness among the pregnant respondents. We calculated survey weights using distributions of age and race (non-Hispanic White, Hispanic, non-Hispanic Black, other) for individuals ages 18–54 reporting a pregnancy between 2010 and 2019 from the National Survey of Family Growth (NSFG) 2017–2019 [24]. Weights were applied in descriptive analyses of changes prenatal care and vaccine willingness to obtain parameters for a U.S. pregnant population. We described changes in prenatal care services, perceived quality of care, and birth plans as well as availability of resources, including overall numbers and weighted percentages (unweighted analyses are specified). We conducted a multivariable logistic regression model to examine associations between vaccine willingness and participant characteristics among the pregnant women. We also presented reasons for not wanting to receive a COVID-19 vaccine and most trusted source of information about COVID-19 vaccines. Willingness to receive a COVID-19 vaccine was collapsed to combine willing and extremely willing responses and unwilling and extremely unwilling responses for most analyses.

To assess how pregnancy is associated with willingness to receive COVID-19 vaccination, we matched the pregnant women to three non-pregnant women controls using propensity score matching. Propensity score matching was done in R (v 4.0.3) and R Studio (v 1.2.5033) using the MatchIt package (v 4.1.0) [25]. Matching was conducted after restricting the sample of complete, eligible survey responses (*n* = 8481) to women of ages 18–54 (*n* = 2909). Nearest neighbor matching with a ratio of 3 non-pregnant controls to 1 pregnant woman was used. Variables matched on included age (18–24, 25–34, 35–44, 45–54), race (non-Hispanic White, non-Hispanic Black, Hispanic, other), education (high school or less, associate degree, some college, bachelor’s degree, graduate degree), income (<$20 k, $20-$39 k, $40-$69 k, $70-$99 k, $100-$149 k, $150 k+), health insurance (no, yes), chronic conditions (0, ≥1), and political affiliation (Republican, Democrat, independent, other). There were no significant differences between pregnant women and non-pregnant controls on matched variables. Among the 932 pregnant women and non-pregnant controls, we conducted multivariable conditional logistic regression models to examine associations between pregnancy status and willingness to receive a COVID-19 vaccine adjusted for participant characteristics. Statistical analyses were conducted in Stata 16.1 (StataCorp, College Station, Texas, USA).

### Ethical approval and consent

This research was performed in accordance with the Declaration of Helsinki. Participants provided electronic consent to participate by responding to a question on the survey. The study received ethical approval from the Institutional Review Board at Johns Hopkins Bloomberg School of Public Health, Baltimore, USA (IRB00012413).

## Results

The Johns Hopkins University National Pandemic Pulse survey was distributed to 10,107 respondents; complete responses were obtained from 8481 participants (83.9%), of which 233 participants were pregnant. Survey respondents included 233 pregnant women (Table 1). Over one-third (*n* = 85, 36.5%) were ages 18–24, 41.2% (*n* = 96) 25–34, and 22.3% (*n* = 52) 35–55 (unweighted). Half were Hispanic (*n* = 116, 49.8%), 25.3% Non-Hispanic White (*n* = 59), and 18.9% Non-Hispanic Black (*n* = 44) (unweighted). Two-thirds were in their third trimester (*n* = 135, 68.5%), a quarter in the second (*n* = 48, 24.4%), and the remainder in the first (*n* = 14, 7.1%) (unweighted).

**Table 1:** Characteristics of pregnant women

Characteristic^a^	Unweighted No. (%) ***n*** = 233	Weighted %
**Age**		
18–24	85 (36.5)	26.6
25–34	96 (41.2)	56.6
35–54	52 (22.3)	16.8
**Race**		
Non-Hispanic white	59 (25.3)	53.0
Non-Hispanic black	44 (18.9)	16.3
Hispanic	116 (49.8)	21.2
Other	14 (6.0)	9.6
**Education**		
High school or less	61 (26.2)	28.8
Associate degree	37 (15.9)	13.1
Some college	35 (15.0)	15.1
Bachelor’s degree	57 (24.5)	24.9
Graduate degree	43 (18.5)	18.0
**Region**		
Midwest	41 (17.6)	23.2
Northeast	37 (15.9)	17.2
South	94 (40.3)	37.3
West	61 (26.2)	22.4
**Income**		
< 20 K	39 (17.0)	16.8
20-39 K	58 (25.3)	25.0
40-69 K	39 (17.0)	17.3
70-99 K	40 (17.5)	16.4
100-149 K	28 (12.2)	13.8
150+	25 (10.9)	10.6
**Lost job or more than half income during pandemic**		
No	119 (51.5)	55.0
Yes	112 (48.5)	45.0
**Health insurance**		
No	42 (18.8)	17.1
Yes	182 (81.2)	82.9
**Chronic conditions**		
0	132 (56.7)	61.9
≥ 1	101 (43.3)	38.1
**Political affiliation**		
Republican	66 (29.9)	35.5
Democrat	105 (47.5)	39.1
Independent or other	50 (22.6)	25.5
**Trimester at survey**		
1st (< 13 wks)	14 (7.1)	8.5
2nd (≥13–26 wks)	48 (24.4)	29.8
3rd (≥26 wks- < 45 wks)	135 (68.5)	61.7

^a^Missing values: income (*n* = 4, 1.7%), lost job (*n* = 2, 0.9%), health insurance (*n* = 9, 3.9%), political affiliation (*n* = 12, 5.2%), trimester (*n* = 36, 15.5%)

### Changes in prenatal care

Forty percent of pregnant women reported that the quality of care they received from their prenatal care providers worsened or remained the same during to the COVID-19 pandemic (*n* = 94, 40.3%); among these women, 12.8% (*n* = 28) said it was significantly worsened (Table 2). The majority, however, reported that the quality of care received from their providers was improved during the pandemic compared to pre-pandemic (*n* = 126, 59.8%). This question was unassociated with participant demographic and socioeconomic characteristics. The most commonly reported changes in prenatal care were change in the format of care (*n* = 84, 34.5%), fewer visits (*n* = 69, 23.8%), and change in provider (*n* = 56, 21.9%); one-quarter of women (*n* = 47, 26.0%) reported no changes in care. Over half of women reported having access to in-person prenatal visits (*n* = 126, 59.5%) and about a third had access to virtual (*n* = 82, 36.6%) and telephone (audio-only) visits (*n* = 76, 37.2%). Many fewer had access to resources such as home care (e.g., blood pressure or fetal heart rate monitoring). Roughly two-thirds of women reported a change in their birth plan related to the COVID-19 pandemic (*n* = 159, 63.5%). The most common reasons for changes to the birth plan were change in planned Cesarean delivery (C-section) (*n* = 50, 19.0%), hospital to home birth (*n* = 45, 17.7%), and vaginal delivery to C-section (*n* = 42, 16.5%).

**Table 2:** Changes in prenatal care and birth planning

Response^a^	No. (weighted %)
**Perceived quality of care**	
Sig. improved	51 (22.8)
Somewhat improved	75 (37.0)
Somewhat worsened, no change	66 (27.4)
Sig. worsened	28 (12.8)
**Change in prenatal care**^b^	
Format of prenatal care	84 (34.5)
Fewer prenatal visits	69 (23.8)
Change in provider	56 (21.9)
None of these	47 (26.0)
Cancelled hospital tour	41 (19.2)
Change to virtual visit	36 (15.6)
**Prenatal resources available**^b^	
In-person visits	126 (59.5)
Virtual visits	82 (36.6)
Phone visits	76 (37.2)
Online messaging	65 (30.6)
Emergency care	47 (24.2)
Home BP monitoring	36 (14.1)
Home fetal heart rate monitoring	21 (9.4)
Don’t know	7 (2.2)
**Change in birth plan**	
Home to hospital	22 (10.2)
Hospital to home	45 (17.7)
Vaginal to C-section	42 (16.5)
Planned C-section changed	50 (19.0)
No changes	66 (36.5)
**Experienced emotional distress about pregnancy**	
No or mild stress	85 (40.3)
Moderate or severe stress	141 (59.7)

^a^Missing values: perceived quality of care (*n* = 13, 5.6%), changes in birth plan (*n* = 8, 3.4%), emotional distress (*n* = 7, 3.0%)

^b^Multiple options allowed

### Willingness of pregnant women to receive COVID-19 vaccination

Forty percent of pregnant women (*n* = 78, 39.8%) were unwilling to receive the COVID-19 vaccine (Table 3). Willingness to receive the COVID-19 vaccine was lower among women in their first trimester (*n* = 2/13, 4.6% (weighted)), compared to those in their second trimester (*n* = 24/45, 44.2%), or third trimester (*n* = 96/134, 68.7%) (*p* < 0.001). In a logistic regression model including demographic variables for age, race, education, income, insurance, chronic disease, region, and political affiliation, only region (Midwest vs. Northeast: aOR 0.10, 95% CI: 0.02, 0.48; South vs. Northeast: aOR 0.13, 95% CI: 0.03, 0.58) and age (35–54 vs. 18–24: aOR 3.99, 95% CI: 1.08, 14.72 were statistically significantly related to the outcome of willing vs. unwilling (Appendix 1). Among pregnant women unwilling to receive the vaccine, leading reasons included concerns about safety (*n* = 40, 54.2%), effectiveness (*n* = 38, 50.1%), long term health concerns (*n* = 29, 43.7%), and conspiracy theories (*n* = 21, 36.3%).

**Table 3:** Willingness to receive COVID-19 vaccine and trusted sources of information

Response^a^	No. (weighted %)
**Willingness to receive COVID-19 vaccine**	
Extr. willing	70 (24.3)
Willing	80 (35.9)
Unwilling	47 (20.0)
Extr. unwilling	31 (19.8)
**Reasons unwilling to receive COVID-19 vaccine**^b^	
Safety	40 (54.2)
Effectiveness	38 (50.1)
Long term health concern	29 (43.7)
Conspiracy theory	21 (36.3)
Do not want to be first	15 (22.7)
Costs concerns	11 (12.8)
Unnecessary	10 (16.7)
Religious reasons	6 (7.1)
Allergy	5 (6.9)
**Most trusted source of information about vaccines**	
CDC	48 (25.6)
Religious groups	2 (1.6)
Federal govt.	3 (1.4)
State/local health dept.	23 (10.9)
Social media	20 (9.1)
News media	22 (8.2)
Family	44 (17.7)
Primary care doctor	60 (25.4)

^a^Missing values: vaccine willingness (*n* = 5, 2.2%), info source (*n* = 11, 4.7%)

^b^Multiple options allowed

Pregnant women’s most trusted sources of information about vaccines were the CDC (*n* = 48, 25.6%), their primary care doctor (*n* = 60, 25.4%), and their family (*n* = 44, 17.7%). The fewest pregnant women responded that religious groups (*n* = 2, 1.6%) or the federal government (*n* = 3, 1.4%) were their most trusted sources of information on vaccines. Those willing to receive a vaccine more often reported that their most trusted source of information about vaccines was their primary care provider (willing: 31.8% vs. unwilling: 17.0%), family (willing: 19.6% vs. unwilling: 15.3%), or social media or news media (willing: 19.1% vs. unwilling: 14.9%), and less often reported the government (willing: 29.6% vs. unwilling: 52.9%) (*p* = 0.062).

### Differences in vaccine willingness between pregnant and non-pregnant women

**Fig. 1:**
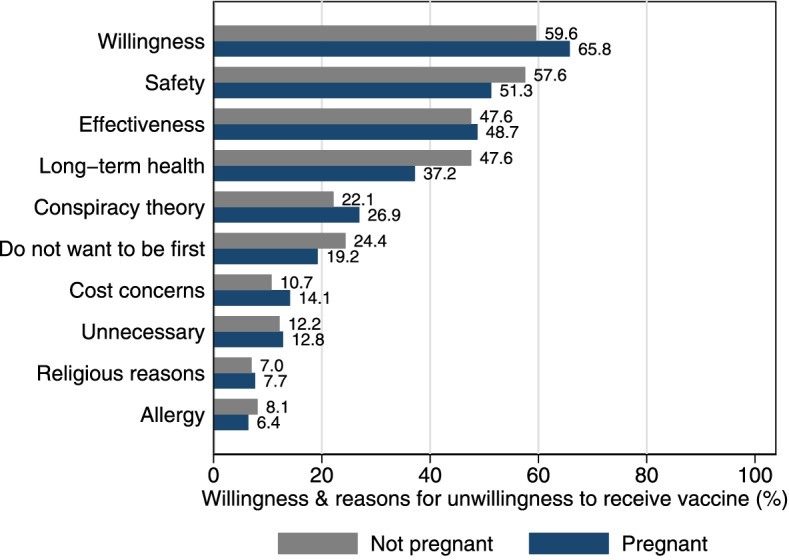
COVID-19 vaccine willingness and reasons for unwillingness to receive the vaccine for pregnant women and non-pregnant matched controls. The percent of pregnant and non-pregnant women who reported that they were willing to receive the COVID-19 vaccine and the percent expressing each concern among those who were unwilling to receive the COVID-19 vaccine

A higher, but not statistically significant, proportion of pregnant women reported being willing to accept a COVID-19 vaccine in a bivariate comparison (pregnant: 65.8% vs. not-pregnant: 59.6% (*p* = 0.098). Reasons for unwillingness to receive a vaccine did not differ between pregnant women and non-pregnant women (Fig. 1) (all *p*-values > 0.1). Pregnant vs. non-pregnant women reported trusted sources for vaccine information, including family (19.8% vs. 13.0%, respectively), social media or news media (18.9% vs. 12.2%, respectively), and the CDC (21.6% vs. 37.3%, respectively) (*p* < 0.001).

In multivariable analysis (Table 4), pregnant women were more likely than non-pregnant women ages 18–54 to express willingness to receive the COVID-19 vaccination, although this did not reach the *p* < 0.05 threshold for statistical significance (*p* = 0.087) (aOR 1.38, 95% CI: 0.95, 2.00). Independent of pregnancy, women with higher education (graduate degree vs. bachelor’s degree: aOR 2.07, CI: 1.06, 4.05) or income (≥$100,000 vs. ≥$40- < $100,000: aOR 1.99, 95% CI: 1.12, 3.51) were more likely to accept vaccination.

**Table 4:** Multivariable logistic conditional regression models for willingness to receive COVID-19 vaccine for pregnant and non-pregnant women

Characteristic	aOR (95% CI) (***n*** = 645)
**Pregnant**	
No	Ref
Yes	1.38 (0.95, 2.00)
**Age**	
18–24	Ref
25–34	1.06 (0.68, 1.68)
35–54	0.92 (0.41, 2.04)
**Race**	
Non-Hispanic White	Ref
Non-Hispanic Black	0.93 (0.47, 1.82)
Hispanic	1.53 (0.72, 3.26)
Other	0.82 (0.32, 2.14)
**Education**	
High school or less	0.78 (0.43, 1.40)
Associate’s degree or some college	0.71 (0.42, 1.22)
Bachelor’s degree	Ref
Graduate degree	**2.07 (1.06, 4.05)**
**Household income**	
< $40,000	0.94 (0.60, 1.47)
$40,000–$99,999	Ref
≥ $100,000	**1.99 (1.12, 3.51)**
**Health insurance**	
No	Ref
Yes	1.43 (0.86, 2.40)
**Chronic conditions**	
0	Ref
≥ 1	1.40 (0.84, 2.34)
**Region**	
Midwest	Ref
Northeast	1.12 (0.59, 2.12)
South	0.87 (0.50, 1.52)
West	0.85 (0.45, 1.59)
**Political party**	
Republican	Ref
Democrat	1.55 (0.91, 2.63)
Independent	0.87 (0.45, 1.69)

*aOR* Adjusted odds ratio

## Discussion

The COVID-19 pandemic changed prenatal care and birth plans for many pregnant women in the United States in 2020. Our study found that most women experienced a change in their prenatal care or birth plans, especially from a planned hospital birth to a home birth or from a vaginal birth to a C-section. Almost half of participants reported that their prenatal care was unaffected or worsened by the pandemic; however, most participants said that their care had improved during the pandemic, reasons for which are unclear. Our study results contribute to the limited evidence on COVID-19 related disruptions in prenatal care, particularly reduced in-person visits and changes in formats [26, 27]. For example, a US online survey of 4451 pregnant women conducted in April to May, 2020, found that almost half (46%) had prenatal visits cancelled or rescheduled [28]. A global survey of maternal and newborn health professionals reported multiple changes in services across countries, including fewer in-person clinic visits, reduced number of visitors permitted, and limited access to educational sessions, which authors suggested could negatively affect quality of care [29]. Our study also found that many women experienced changes in labor and delivery care, especially shifts from planned hospital to home birth or from vaginal to C-section delivery. Previous studies have reported different findings about changes in the incidence of C-section, with some reporting no change [30], decreased rates [31, 32], and others slightly increased rates [33], despite no evidence for a change in the indication for C-section in the context of the COVID-19 pandemic [7, 34]. Such changes in access and quality of prenatal care and labor and delivery services are likely important contributors to emotional stress during pregnancy, a finding observed elsewhere due to the impact of COVID-19 and in the context of previous pandemics [35].

Many women were unwilling to receive the COVID-19 vaccine, with higher rates of unwillingness among those in their first trimester, younger women, and women living in the Midwest and South. However, there was an indication that pregnant women may have been more willing to receive a vaccine than similar women who were not pregnant. The reasons for this are unknown, but this could perhaps be a result of recognition among the pregnant population of their increased risk of severe COVID-19 disease and adverse pregnancy outcomes. Other studies have reported lower rates of vaccine acceptance among pregnant women compared to non-pregnant women, although few have been designed to investigate this question specifically [20, 36]. For example, a survey of pregnant women and mothers of young children across 16 countries in October and November 2020 found that 52% of pregnant women and 73% of non-pregnant women intended to receive the COVID-19 vaccine [20].

Safety is the most commonly reported concern about vaccines among pregnant women, and this issue was also the leading reason cited by women unwilling to receive the COVID-19 vaccine in our survey [37, 38]. A cross-sectional study of three centers in the United States between August and December 2020 reported vaccine safety concerns for their pregnancy (82%) as the leading concern for COVID-19 vaccination among pregnant women, followed by concerns about their own health (68%), vaccine effectiveness (52%), and the belief that the vaccine was not needed (22%) [21]. Among this population, the most trusted sources of COVID-19 information were also health professionals (obstetrician/gynecologist: 42%; family doctor or primary care doctor: 28%; followed by the CDC: 13%) [21]. Pregnant women who were Non-Hispanic Black and Hispanic were less likely than women who were Non-Hispanic White to express willingness to receive the COVID-19 vaccine [21]. This finding has been reported in other US settings [36], although our study was not designed to address this research question. Further research, policy, and programs efforts are needed to address these disparities, particularly given the context of higher risk of serious COVID-19 illness among racial and ethnic minorities and the history of mistreatment and persistent structural racism in medicine and public health [39].

In many contexts, the strongest driver of maternal vaccine acceptance is the recommendation of a health care professional [37]. This is consistent with our finding that primary care providers and the CDC were among pregnant women’s most trusted sources of information. A systematic review and meta-analysis showed that other important drivers of vaccine uptake among pregnant women are vaccine-specific factors and disease-related risk perceptions, including the belief that the vaccine would benefit the mother and fetus and not cause harm [40]. Vaccine effectiveness and belief in conspiracy theories were also prominent reasons for unwillingness to receive the COVID-19 vaccine in our sample. The systematic review did not find clear evidence to link belief in susceptibility to pandemic or seasonal influenza and increased vaccine uptake; however, they found moderate evidence that perceptions of severity of the pandemic and risk of hospitalization were associated with vaccination status [40].

The level of vaccine willingness among pregnant women reported in this study may change in context of the rapidly evolving pandemic, particularly because available COVID-19 vaccines have only been used in healthy adults for a short period of time relative to other maternal vaccines. Further, although CDC, ACOG, and other health professions groups made early recommendations that pregnant and breastfeeding women have access to COVID-19 vaccines, the CDC’s recommendation in August 2021 that these women receive the vaccine could increase vaccine acceptance and uptake [19]. However, this is no guarantee and, in fact, newly issued recommendations also have the potential to generate confusion and increase vaccine hesitancy and refusal if not communicated appropriately [41].

Our study had several limitations. Selection bias associated with online surveys may underrepresent individuals who are older, without internet access, have lower income, and have less formal education; we attempted to account for this bias in part by weighting on age and race to a national population of pregnant women. Despite the large sample size of our full survey, the number of pregnant respondents was too small to rigorously examine our research questions stratified by race/ethnicity, income, and education. Our questionnaire was cross-sectional at a single time point and did not collect data on reasons for changes in prenatal care, emotional distress related to pregnancy, or acceptance of vaccination, constraining conclusions regarding the drivers of experiences faced by pregnant women in the context of the COVID-19 pandemic. Further, as this survey was designed to rapidly investigate research questions at multiple time points in the pandemic, we were not able to assess the validity or reliability of the instrument. Our study asked only about willingness to receive a COVID-19 vaccine but did not assess actual vaccination status because the survey was implemented before these vaccines were widely available to the public.

The study has direct implications for public health practice. To support pregnant women through the perinatal care continuum, maternity care teams, and professional associations more broadly, should develop protocols to foster social support, patient-centered education around infection prevention that focuses on improved risk perception, expected changes in care due to COVID-19, and vaccine effectiveness and safety. In addition to research on clinical aspects of COVID-19 in pregnancy, further study is needed to understand the impact of this pandemic on care quality including racial/ethnic disparities, development and testing of COVID-19 appropriate perinatal counselling, and evaluation of telehealth care models to improve perinatal care and support services.

## Supplementary Information

**Additional file 1:** Multivariable logistic regression models for willingness to receive COVID-19 vaccine among pregnant women by participant characteristics.

**Additional file 2.**

## Data Availability

The datasets used and analyzed in the current study are available on reasonable request. Please contact Dr. Alain Labrique (alabriq1@jhu.edu).

## References

[1] Peahl AF, Smith RD, Moniz MH (2020). Prenatal care redesign: creating flexible maternity care models through virtual care. Am J Obstet Gynecol.

[2] Davis-Floyd R, Gutschow K, Schwartz DA (2020). Pregnancy, birth and the COVID-19 pandemic in the United States. Med Anthropol.

[3] Kaiser Permanente. 2021. https://thrive.kaiserpermanente.org/care-near-you/northern-california/.

[4] Zambrano LD, Ellington S, Strid P, Galang RR, Oduyebo T, Tong VT (2020). Update: characteristics of symptomatic women of reproductive age with laboratory-confirmed SARS-CoV-2 infection by pregnancy status - United States, January 22-October 3, 2020. MMWR Morb Mortal Wkly Rep.

[5] Chmielewska B, Barratt I, Townsend R, Kalafat E, van der Meulen J, Gurol-Urganci I, et al. Effects of the COVID-19 pandemic on maternal and perinatal outcomes: a systematic review and meta-analysis. Lancet Glob Health. 2021. 10.1016/S2214-109X(21)00079-6. 10.1016/S2214-109X(21)00079-6 PMC8012052 33811827

[6] Mullins E, Hudak ML, Banerjee J, Getzlaff T, Townson J, Barnette K (2021). Pregnancy and neonatal outcomes of COVID-19: coreporting of common outcomes from PAN-COVID and AAP-SONPM registries. Ultrasound Obstet Gynecol.

[7] Di Girolamo R, Khalil A, Rizzo G, Capannolo G, Buca D, Liberati M, et al. Systematic review and critical evaluation of quality of clinical practice guidelines on the management of SARS-CoV-2 infection in pregnancy. Am J Obstet Gynecol MFM. 2022;4(5):100654. 10.1016/j.ajogmf.2022.100654. Epub ahead of print. 10.1016/j.ajogmf.2022.100654 PMC9057927 35504493

[8] Gray KJ, Bordt EA, Atyeo C, Deriso E, Akinwunmi B, Young N, et al. COVID-19 vaccine response in pregnant and lactating women: a cohort study. Am J Obstet Gynecol. 2021. 10.1016/j.ajog.2021.03.023. 10.1016/j.ajog.2021.03.023 PMC7997025 33775692

[9] Rottenstreich A, Zarbiv G, Oiknine-Djian E, Zigron R, Wolf DG, Porat S (2021). Efficient maternofetal transplacental transfer of anti- SARS-CoV-2 spike antibodies after antenatal SARS-CoV-2 BNT162b2 mRNA vaccination. medRxiv.

[10] Mithal LB, Otero S, Shanes ED, Goldstein JA, Miller ES. Cord blood antibodies following maternal coronavirus disease 2019 vaccination during pregnancy. Am J Obstet Gynecol. 2021. 10.1016/j.ajog.2021.03.035. 10.1016/j.ajog.2021.03.035 PMC8012273 33812808

[11] Male V (2021). Are COVID-19 vaccines safe in pregnancy?. Nat Rev Immunol.

[12] Prasad S, Kalafat E, Blakeway H, Townsend R, O’Brien P, Morris E (2022). Systematic review and meta-analysis of the effectiveness and perinatal outcomes of COVID-19 vaccination in pregnancy. Nat Commun.

[13] Zauche LH, Wallace B, Smoots AN, Olson CK, Oduyebo T, Kim SY, et al. Receipt of mRNA COVID-19 vaccines preconception and during pregnancy and risk of self-reported spontaneous abortions, CDC v-safe COVID-19 vaccine pregnancy registry 2020-21. Res Square. 2021. 10.21203/rs.3.rs-798175/v1.

[14] Shimabukuro TT, Kim SY, Myers TR, Moro PL, Oduyebo T, Panagiotakopoulos L (2021). Preliminary findings of mRNA Covid-19 vaccine safety in pregnant persons. N Engl J Med.

[15] Rasmussen SA, Jamieson DJ (2021). Pregnancy, postpartum care, and COVID-19 vaccination in 2021. JAMA.

[16] Centers for Disease Control and Prevention (2021). Information about COVID-19 vaccines for people who are pregnant or breastfeeding.

[17] American College of Obstetricians and Gynecologists (2021). Vaccinating Pregnant and Lactating Patients Against COVID-19.

[18] Society for Maternal-Fetal Medicine (2021). Provider considerations for engaging in COVID-19 vaccine counseling with pregnant and lactating patients.

[19] Centers for Disease Control and Prevention (2021). COVID-19 vaccines while pregnant or breastfeeding.

[20] Skjefte M, Ngirbabul M, Akeju O, Escudero D, Hernandez-Diaz S, Wyszynski DF (2021). COVID-19 vaccine acceptance among pregnant women and mothers of young children: results of a survey in 16 countries. Eur J Epidemiol.

[21] Battarbee AN, Stockwell MS, Varner M, Newes-Adeyi G, Daugherty M, Gyamfi-Bannerman C (2022). Attitudes toward COVID-19 illness and COVID-19 vaccination among pregnant women: a cross-sectional multicenter study during august-December 2020. Am J Perinatol.

[22] Feinberg E. Shattering the infertility myth: what we know about Covid-19 vaccines and pregnancy. STAT. 2021. Available at: https://www.statnews.com/2021/03/25/infertility-myth-covid-19-vaccines-pregnancy/. Accessed 13 May 2021.

[23] Environmental Influences on Child Health Outcomes (ECHO) program. COVID-19 Questionnaire – Adult Primary Version. https://www.nlm.nih.gov/dr2/C19-aPV_COVID-19_Questionnaire-Adult_Primary_Version_20200409_v01.30.pdf.

[24] CDC National Center for Health Statistics. National survey of family growth 2017–2019. Hyattsville: U.S. Department of Health and Human Services, Centers for Disease Control and Prevention, National Center for Health Statistics; 2020.

[25] Austin PC (2011). An introduction to propensity score methods for reducing the effects of confounding in observational studies. Multivariate Behav Res.

[26] Claudio E, Donahue J, Niles PM, Pirsch A, Ramos P, Neely I (2020). Mobilizing a public health response: supporting the perinatal needs of new Yorkers during the COVID-19 pandemic. Matern Child Health J.

[27] Kotlar B, Gerson E, Petrillo S, Langer A, Tiemeier H (2021). The impact of the COVID-19 pandemic on maternal and perinatal health: a scoping review. Reprod Health.

[28] Preis H, Mahaffey B, Heiselman C, Lobel M (2020). Vulnerability and resilience to pandemic-related stress among U.S. women pregnant at the start of the COVID-19 pandemic. Soc Sci Med.

[29] Semaan A, Audet C, Huysmans E, Afolabi B, Assarag B, Banke-Thomas A, et al. Voices from the frontline: findings from a thematic analysis of a rapid online global survey of maternal and newborn health professionals facing the COVID-19 pandemic. BMJ Global Health. 2020;5(6):e002967. 10.1136/bmjgh-2020-002967 PMC7335688 32586891

[30] Malhotra Y, Miller R, Bajaj K, Sloma A, Wieland D, Wilcox W (2020). No change in cesarean section rate during COVID-19 pandemic in new York City. Eur J Obstet Gynecol Reprod Biol.

[31] Einarsdóttir K, Swift EM, Zoega H (2021). Changes in obstetric interventions and preterm birth during COVID-19: a nationwide study from Iceland. Acta Obstet Gynecol Scand..

[32] Li M, Yin H, Jin Z, Zhang H, Leng B, Luo Y (2020). Impact of Wuhan lockdown on the indications of cesarean delivery and newborn weights during the epidemic period of COVID-19. Plos One.

[33] Bhatia K, Columb M, Bewlay A, Eccles J, Hulgur M, Jayan N (2021). The effect of COVID-19 on general anaesthesia rates for caesarean section. A cross-sectional analysis of six hospitals in the north-west of England. Anaesthesia.

[34] Di Mascio D, Buca D, Berghella V, Khalil A, Rizzo G, Odibo A (2021). Counseling in maternal-fetal medicine: SARS-CoV-2 infection in pregnancy. Ultrasound Obstet Gynecol.

[35] Choi KR, Records K, Low LK, Alhusen JL, Kenner C, Bloch JR (2020). Promotion of maternal–infant mental health and trauma-informed care during the COVID-19 pandemic. J Obstet Gynecol Neonatal Nurs.

[36] Sutton D, D’Alton M, Zhang Y, Kahe K, Cepin A, Goffman D (2021). COVID-19 vaccine acceptance among pregnant, breastfeeding, and nonpregnant reproductive-aged women. Am J Obstet Gynecol MFM.

[37] Lutz CS, Carr W, Cohn A, Rodriguez L (2018). Understanding barriers and predictors of maternal immunization: identifying gaps through an exploratory literature review. Vaccine.

[38] Wilson RJ, Paterson P, Jarrett C, Larson HJ (2015). Understanding factors influencing vaccination acceptance during pregnancy globally: a literature review. Vaccine.

[39] Muñoz-Price LS, Nattinger AB, Rivera F, Hanson R, Gmehlin CG, Perez A (2020). Racial disparities in incidence and outcomes among patients with COVID-19. JAMA Netw Open.

[40] Kilich E, Dada S, Francis MR, Tazare J, Chico RM, Paterson P (2020). Factors that influence vaccination decision-making among pregnant women: a systematic review and meta-analysis. Plos One.

[41] Rabin RC. C.D.C. recommends Covid vaccines during pregnancy. New York: The New York Times; 2021. Available at: https://www.nytimes.com/2021/08/11/health/covid-vaccine-pregnancy-cdc.html. Accessed 30 Aug 2021.

