# Supplementary material for: Changes in prenatal care and vaccine willingness among pregnant women during the COVID-19 pandemic
Source: BMC Pregnancy Childbirth. 2022 Jul 13;22:558. doi: 10.1186/s12884-022-04882-x (PMC9281008; doi:10.1186/s12884-022-04882-x)
Supplement: Supplementary file 1 — Additional file 1: Multivariable logistic regression models for willingness to receive COVID-19 vaccine among pregnant women by participant characteristics. [file 12884_2022_4882_MOESM1_ESM.docx]

**Multivariable logistic regression models for willingness to receive COVID-19 vaccine among pregnant women by participant characteristics**

| **Characteristic** | **aOR (95% CI) (n=209)** |
| --- | --- |
| **Age** |  |
| 18-24 | Ref |
| 25-34 | 1.58 (0.66, 3.81) |
| 35-54 | 3.99 (1.08, 14.72) |
| **Race** |  |
| Non-Hispanic White | Ref |
| Non-Hispanic Black | 1.39 (0.40, 4.90) |
| Hispanic | 1.66 (0.62, 4.42) |
| Other | 0.56 (0.12, 2.54) |
| **Education** |  |
| High school or less | 0.70 (0.20, 2.48) |
| Associate's degree or some college | 0.65 (0.20, 2.12) |
| Bachelor's degree | Ref |
| Graduate degree | 1.01 (0.26, 3.93) |
| **Household income** |  |
| <$40,000 | 0.93 (0.35, 2.44) |
| $40,000-$99,999 | Ref |
| ≥$100,000 | 2.62 (0.72, 9.46) |
| **Health insurance** |  |
| No | Ref |
| Yes | 0.35 (0.10, 1.26) |
| **Chronic conditions** |  |
| 0 | Ref |
| ≥1 | 1.72 (0.76, 3.89) |
| **Region** |  |
| Midwest | 0.10 (0.02, 0.48) |
| Northeast | Ref |
| South | 0.13 (0.03, 0.58) |
| West | 0.24 (0.04, 1.32) |
| **Political party** |  |
| Republican | Ref |
| Democrat | 1.64 (0.59, 4.51) |
| Independent | 0.62 (0.19, 2.10) |
|  |  |
| aOR: adjusted odds ratio. These are weighted results. | |
