# Supplementary material for: Changes in prenatal care and vaccine willingness among pregnant women during the COVID-19 pandemic
Source: BMC Pregnancy Childbirth. 2022 Jul 13;22:558. doi: 10.1186/s12884-022-04882-x (PMC9281008; doi:10.1186/s12884-022-04882-x)
Supplement: Supplementary file 2 — Additional file 2. [file 12884_2022_4882_MOESM2_ESM.pdf]

PandemicPulse Round2

Welcome to the PandemicPulse Survey

The Johns Hopkins Bloomberg School of Public Health is conducting research about how the COVID-19 pandemic has impacted people's lives. We are asking you to complete a 10-15 minute survey. Responding to this survey request is voluntary; it is your choice. If you complete and submit this survey, we will know that you consent to participate in this study. You may choose not to answer any question that we ask. Your individual responses will not be shared, but we will share grouped results. Thank you for considering participation in our study.

If you have any questions, you may contact us at pulsesurvey@jhu.edu

Click 'NEXT' if you would like to take the survey.

PandemicPulse Round2

Welcome to the PandemicPulse Survey

* 1. By clicking 'Yes' I consent to participate.

- ◯ Yes

PandemicPulse Round2

We would first like you to provide some information about yourself.

* 2. What is your age?

- Under 18
- 18-24
- 25-34
- 35-44
- 45-54
- 55-64
- 65+

* 3. What gender do you identify with?

- ◯ Female
- ◯ Male
- ◯ Other

Other (specify)

PandemicPulse Round2

* 4. Are you currently **pregnant**?

- Yes
- No

* 5. Is this your first pregnancy?

- Yes
- No

* 6. Do you have any of the following conditions? (Select all that apply)

- [ ] Hypertension
- [ ] Diabetes mellitus
- [ ] Cancer/ malignancy
- [ ] Cardiovascular disease
- [ ] Chronic lung disease
- [ ] Chronic liver disease
- [ ] Cerebrovascular disease
- [ ] Chronic kidney disease
- [ ] Immunocompromised state (weakened immune system)
- [ ] Obesity
- [ ] Sickle cell disease
- [ ] No, I do not have any of these conditions

PandemicPulse Round2

* 7. Are you of Hispanic, Latino, or Spanish origin, such as Mexican, Puerto Rican or Cuban?

- Yes
- No
- I don't know
- Prefer not to say

* 8. What is the **primary race/ethnicity** you identify with?

- American Indian or Alaskan Native
- Asian or Pacific Islander
- Black or African American
- Hispanic or Latino
- White/Caucasian
- Prefer not to say
- Other

* 9. What is the **highest level of school** you have completed or the highest degree you have received?

- Less than high school degree
- High school degree or equivalent (e.g., GED)
- Some college but no degree
- Associate degree
- Bachelor degree
- Graduate degree

* 10. Which of the following best describes your current employment status?

- Working full time
- Working part time
- Unemployed or laid off and seeking employment
- Unemployed or laid off, not seeking employment
- Temporarily furloughed
- Hours reduced
- Keeping house or raising children full time
- Retired
- Full-time student

Other (please specify)

* 11. In what state or U.S. territory do you live?

* 12. Do you currently participate in a social bubble or quarantine pod (in which 2-3 households agree to socialize with one another but no one else)?

- Yes
- No
- Prefer not to say

The next few questions are about your experiences with COVID-19

* 13. Since the pandemic started in March 2020, have you EVER wanted or needed to get a test for COVID-19 because you either thought you were exposed or had symptoms?

- ◯ Yes
- ◯ No
- ◯ Prefer not to say

* 14. When did you last need a COVID-19 test?

* 15. Were you able to get tested for COVID-19?

- Yes
- No
- Prefer not to say

* 16. What is the main reason you didn't get tested?

- ◯ Afraid to get tested
- ◯ Didn't know where to go
- ◯ Testing center too far
- ◯ Couldn't get an order from a doctor to get tested
- ◯ Language barriers
- ◯ Too long of a waiting line to get tested
- ◯ Other reasons
- ◯ Prefer not to say

* 17. **How long** did you wait from the time you needed / wanted a COVID-19 test to the time you got one?

- Same day
- 1-2 days
- 3-5 days
- 6-7 days
- More than 1 week
- Prefer not to say

* 18. **How long** did it take you to get your COVID-19 test results?

- ◯ Same day
- ◯ 1-2 days
- ◯ 3-5 days
- ◯ 6-7 days
- ◯ More than 1 week
- ◯ Didn't receive test results
- ◯ Prefer not to say

Other (please specify)

[ ]

* 19. Did you test **positive** for COVID-19?

- Yes, and I had symptoms
- Yes, and I had no symptoms
- No
- Prefer not to say

* 20. Were you hospitalized after you tested positive for COVID-19?

- Yes
- No
- Prefer not to say

PandemicPulse Round2

* 21. Has anyone of your friends or family tested positive with novel coronavirus/COVID-19?

- Yes
- No
- I don't know
- Prefer not to say

The next set of questions will be about vaccines to prevent COVID-19?

* 24. COVID-19 vaccines made by Pfizer/ BioNTech, Moderna, and Astra Zeneca have passed phase 3 clinical trials which showed they are safe and effective in preventing COVID-19. How **willing** would you be to get one of these COVID-19 vaccines?

- ○ Extremely willing
- ○ Willing
- ○ Not willing
- ○ Extremely not willing
- ○ Prefer not to say

* 25. Why would you not be willing to get the vaccine? (Select all that apply)

- ☐ I do not trust the COVID-19 vaccine to be effective
- ☐ I do not trust the COVID-19 vaccine to be safe
- ☐ I am concerned about the financial cost of vaccine
- ☐ I have religious objections to vaccines
- ☐ I am concerned that the vaccine may affect my long-term health
- ☐ I am concerned that the vaccine may be part of a conspiracy
- ☐ I have had allergic reactions to vaccines in the past
- ☐ I may be willing to get the vaccine, but would not want to be one of the first to receive it
- ☐ The vaccine is unnecessary
- ☐ Prefer not to say
- ☐ Other (please specify)

* 26. If we had a limited number of vaccine doses for COVID-19, who do you think should get it **first**?

- Elderly
- Long-term care facility residents
- Health care workers
- Other essential workers (those who are required to physically go to work, e.g., postal service, firefighters, police, etc.)
- People who can pay for it
- People with health conditions
- First come, first served
- Children
- I don't know
- Prefer not to say

* 27. Which of these sources do you trust the most for information about vaccines?

- Your family
- Your primary care doctor
- The news media
- Social Media (Facebook, Twitter etc.)
- State/Local health department
- Centers for Disease Control and Prevention (CDC)
- Religious groups
- Federal government (Executive Branch)
- Prefer not to say

* 28. How likely are you to receive the flu vaccine for this current flu season (2020-2021)?

- Received already
- Extremely likely
- Likely
- Not likely
- Extremely not likely
- Prefer not to say

The next few questions are about things you might have done to protect yourself and your family from COVID-19 in the past month.

* 29. In the **past month**, have you delayed getting medical care (including dental, physical or mental health visits)?

- ◯ Yes
- ◯ No
- ◯ I didn't need to go
- ◯ Prefer not to say

Other (please specify)

* 30. What were the reasons you delayed getting medical attention? Select all that apply

- [ ] Cost
- [ ] Unable to get time away from work
- [ ] Inability to find childcare
- [ ] Concern over exposure to coronavirus (COVID-19)
- [ ] Caring for family member

Other (please specify)

* 31. In the past month, have you avoided getting a medical prescription refilled because you didn't want to leave your house due to COVID-19?

- ◯ Yes
- ◯ No
- ◯ I did not need a refill
- ◯ Prefer not to say

* 32. Since March 2020, have you started taking any medications or supplement that you believe will protect you from COVID-19?

- ◯ Yes
- ◯ No
- ◯ Prefer not to say

PandemicPulse Round2

The next section of this questionnaire will focus on the impacts of COVID-19 on your job and income.

* 34. Have you lost your job or more than half your income due to COVID-19?

- ◯ Yes
- ◯ No
- ◯ Prefer not to say

* 35. How concerned are you that you will be unable to cover the basic costs of your household?

- ◯ very concerned
- ◯ concerned
- ◯ unconcerned
- ◯ very unconcerned
- ◯ Prefer not to say

* 36. When do you worry that you will be unable to cover the basic costs of your household?

- ◯ Immediately
- ◯ In the next 2 weeks
- ◯ In the next month
- ◯ In the next 3 months
- ◯ Prefer not to say

* 37. Since the beginning of the pandemic, was there a time when you couldn’t do one of the following due to cost considerations? Check all that apply.

- ☐ Skipped filling a medical prescription
- ☐ Skipped a medical test recommended by a doctor
- ☐ Skipped a treatment recommended by a doctor
- ☐ Skipped a follow-up recommended by a doctor
- ☐ Had a medical problem but DID NOT go to a doctor or clinic
- ☐ Did not see a specialist when you or your doctor thought you needed one
- ☐ Delayed or did not get dental care
- ☐ Delayed or did not get vision care
- ☐ None of the above
- ☐ Prefer not to say

PandemicPulse Round2

Additional questions for pregnant women

The next few questions are about how COVID-19 has affected activities surrounding your pregnancy.

* 57. When is your due date?

Date / Time

Date

MM/DD/YYYY

* 58. What resources are currently available to you from your prenatal provider (check all that apply)

- [ ] Regular in-person appointments
- [ ] Virtual care appointments
- [ ] Phone call appointments
- [ ] Online messaging portal for questions/concerns
- [ ] Emergency care
- [ ] Home blood pressure monitoring
- [ ] Home fetal heart rate monitoring
- [ ] I don't know

Other (please specify)

* 59. Which of the following changes are you experiencing in your prenatal care as a result of the COVID-19 outbreak? (Check all that apply).

- [ ] Change in prenatal care provider
- [ ] Cancellation of or reduction in frequency of prenatal visit(s)
- [ ] Changed format of prenatal care (e.g. no group classes)
- [ ] Cancellation of hospital tours
- [ ] Transition from in-person prenatal to virtual visits
- [ ] None apply

* 60. In general, how distressed are you about changes to your prenatal care due to the COVID-19 outbreak?

- Not at all
- Mildly
- Moderately
- Extremely
- Prefer not to say

* 61. How has the support you receive from your prenatal care provider(s) changed due to the COVID-19 outbreak?

- Significantly worsened
- Somewhat worsened, no change
- Somewhat improved
- Significantly improved
- Prefer not to say

* 62. Which of the following changes are your experiencing in your birth plan as a result of the COVID-19 outbreak?

- I changed from planning a vaginal birth to a C-section
- My planned C-section or labor induction was changed
- I changed from planning a hospital birth to a home birth
- I changed from planning a home birth to a hospital birth
- No changes
- Prefer not to say

* 63. What is your current annual household income from all sources?

- Less than 10,000
- 10,000 to 19,999
- 20,000 to 29,999
- 30,000 to 39,999
- 40,000 to 49,999
- 50,000 to 69,999
- 70,000 to 84,999
- 85,000 to 99,999
- 100,000 to 149,999
- 150,000 to 199,999
- 200,000 or more
- Prefer not to say

* 64. In politics TODAY, do you consider yourself a Republican, Democrat, or independent?

- Republican
- Democrat
- Independent
- Other party
- Prefer not to say

* 65. Are you covered by any kind of health insurance or some other kind of health care plan?

- Yes
- No
- I don't know
- Prefer not to say

PandemicPulse Round2

* 66. What type of health insurance or health care coverage do you have?

- ◯ Insurance through a current or former employer or union (by you or another family member)
- ◯ Insurance purchased from an insurance company (by you or another family member)
- ◯ Medicare, for people 65 and older, or people with certain disabilities
- ◯ Medicaid, Medical Assistance, or any government-assistance plan for those with low incomes or a disability
- ◯ TRICARE or other military healthcare
- ◯ VA (enrolled for VA healthcare)
- ◯ Indian Health Service
- ◯ I don't know
- ◯ Any other type of health insurance or health coverage plan (Please Describe)

Other (please specify)

* 67. Since March 13, 2020 have you applied for Unemployment Insurance (UI) benefits?

- Yes
- No
- Prefer not to say
